# Negative Differential Conductance Assisted by Optical Fields in a Single Quantum Dot with Ferromagnetic Electrodes

**DOI:** 10.3390/nano9060863

**Published:** 2019-06-06

**Authors:** Weici Liu, Faqiang Wang, Zhilie Tang, Ruisheng Liang

**Affiliations:** 1 Guangdong Research Center of Photoelectric Detection Instrument Engineering Technology, and Guangdong Laboratory of Quantum Engineering and Quantum Materials, School of physics and Telecommunication Engineering, South China Normal University, Guangzhou 510006, China; liuweici-2002@126.com; 2 Department of Electronic Information Engineering, Guangzhou College of Technology and Business, Foshan 528138, China; 3 Guangzhou Key Laboratory for Special Fiber Photonic Devices, Laboratory of Nanophotonic Functional Materials and Devices, School of Information and Optoelectronic Science and Engineering, South China Normal University, Guangzhou 510006, China; fqwang@scnu.edu.cn (F.W.); gdz01@scnu.edu.cn (R.L.)

**Keywords:** quantum dot, ferromagnetic electrodes, negative differential conductance, Keldysh nonequilibrium Green’s function, optical fields

## Abstract

In a single quantum dot (QD) system connected with ferromagnetic electrodes, the electron transport properties, assisted by the thermal and Fock state optical fields, are theoretically studied by the Keldysh nonequilibrium Green’s function approach. The results show that the evolution properties of the density of state and tunneling current assisted by the Fock state optical field, are quite different from those of the thermal state. The photon sideband shift decreases monotonously with the increase in the electron–photon coupling strength for the case of the thermal state, while the shift is oscillatory for the case of the Fock state. Negative differential conductance (NDC) appears obviously in a QD system contacted with parallel (*P*) and antiparallel (*AP*) magnetization alignment of the ferromagnetic electrode leads, assisted by the Fock state optical field in a wide range of electron–photon interaction parameters. Evident NDC usually only arises in an *AP* configuration QD system assisted by the thermal state optical field. The results have the potential to introduce a new way to actively manipulate and control the single-electron tunneling transport on a QD system by the quantum states of the optical field.

## 1. Introduction

Circuit quantum electrodynamics (QED) enable people to manipulate and probe with high sensitivity the quantum state of superconducting quantum bits coupled to microwave cavities. Recently, it has become possible to fabricate new devices, in which the superconducting quantum bits are replaced by hybrid mesoscopic circuits, combining nanostructure devices and metallic reservoirs. Owing to the versatility of nanofabricated circuits, the hybrid circuit QED would be suitable for a number of applications which are not accessible with standard cavity QED [1]. 

Because of the discrete energy spectrum and behavior similar to that of an atom [2], a quantum dot (QD)-based hybrid circuit, QED, could be used to probe the interactions of light with matter (light–matter interactions) [3,4,5,6], to implement the quantum optical device [7,8], in order to engineer new states of matter with relevance to the fields of quantum optics [9,10] and solid state physics [11,12]. 

The greatest advantage of the hybrid circuit QED systems is that the artificial atom properties can be arbitrarily controlled by the application of an external field. It provides a new way to study the light–matter interactions in electronic circuits. There are mainly two kinds of studies. The first class of studies focuses on the artificial atom limit, where the hybrid circuit QED can be used to manipulate and probe the electronic degrees of freedom, such as confined charges or spins. The second class of studies focuses on revealing or controlling the dynamics of electron tunneling between QD and electrode leads using a cavity photon field.

On the other hand, negative differential conductance (NDC) has attracted considerable attention due to its potential applications in the realization of low-power memory devices and logic circuits. NDC could be used to reveal the intrinsic highly nonlinear character of molecular junctions, and it appears to be related to fundamental features of electron–electron or electron–vibration interactions [13,14]. The typical system, where NDC appears, is a QD with a single orbital level, coupled to an on-site single phonon mode, and connected to leads with asymmetric tunneling rates [15]. NDC can also be found in phonon-assisted QD systems, with finite Coulomb correlation parameters, connected to an asymmetric magnetization alignment of the ferromagnetic electrode leads [16]; a double QDs system connected to symmetric or asymmetric magnetization alignment of the ferromagnetic electrodes leads [17]; and a molecular junction connected to leads with asymmetric tunneling rate [18]. In the past, the phonons, in all the phonon-assisted QD systems investigating NDC, are in a thermal state, and it is difficult to actively modulate and control the NDC. It is interesting to investigate how other quantum states influence the dynamics of electron tunneling via the interaction of a QD with another form of external field, especially the NDC property.

In this paper, the purpose is to investigate the dynamics of electron tunneling through a photon-assisted QD system connected to the ferromagnetic electrodes leads with an infinite Coulomb correlation parameter. The QD system can be implemented by the hybrid circuit QED technique, where the dynamics of electron tunneling can be controlled by the cavity photon field. The state of the assisting photon field in this paper is the Fock state, and the results are compared with that of the thermal state.

Photon-assisted electronic transport attracts great attention [19,20]. However, most of the studies employ a classical treatment for the external field, that introduces a time-dependent oscillating energy level in QD, which is valid only in the case of a high-intensity field and weak coupling [21]. It has been shown that the spectrum in the quantum case is shifted from the non-interacting spectrum, and that the shift of photon sidebands is photon-intensity-dependent [21,22]. Employing the quantum treatment of the electron–photon interaction [21] and the Keldysh non-equilibrium Green’s function approach [23,24,25,26], we study photon-assisted transport properties of electrons through a single QD connected with ferromagnetic electrode leads.

The paper is organized as follows. In Section 2, we present the physical model and theoretical calculation by the Keldysh non-equilibrium Green’s function approach, based on the quantum treatment of the optical field. In Section 3, we study the effects of the electron–photon coupling strength, bias voltage and other parameters of optical fields on the density of states and tunneling current. The conclusion will be given in Section 4.

## 2. Physical Model and Formalism

**Figure 1:**
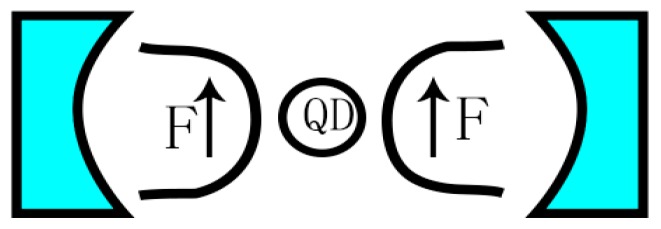
The system structure sketch.

Figure 1 illustrates the system schematic of the QD with ferromagnetic leads coupled to a one-mode optical cavity; the QD is modeled as a one-level system. The loss of the optical field is not considered here, because we focus on the influence of the thermal and Fock state optical fields on the transport properties through a single QD.

The total Hamiltonian of the system can be written as *H* = *H_L_* + *H_R_* + *H_ph_* + *H_D_* + *H_T_*. The Hamiltonians for electrons in the left (L) and right (R) electrode leads are [1,8,21],

(1) $$H_{L}+H_{R}=\sum_{k\sigma ,\alpha \in L,R}\varepsilon_{k\sigma ,\alpha }c_{k\sigma ,\alpha }^{†}c_{k\sigma ,\alpha }$$

where $c_{k\sigma ,\alpha }^{†}(c_{k\sigma ,\alpha })$ is the conduction electron creation (annihilation) operator with wave vector *k* and spin *σ* in lead α, and $\varepsilon_{k\sigma ,\alpha }$ is the spin-dependent single-electron energy.

The third term stands for the single-mode optical field. $H_{ph}=\hbar \omega_{0}a^{†}a$, where $a^{†}\left(a\right)$ is the photon creation (annihilation) operator with frequency $\omega_{0}$. The electron Hamiltonian on the QD is:

(2) $$H_{D}=\sum_{\sigma }\left[\varepsilon_{\sigma }+eV_{g}+\lambda (a^{†}+a)\right]d_{\sigma }^{†}d_{\sigma }^{}+Ud_{↑}^{†}d_{↑}d_{↓}^{†}d_{↓}$$

where $\varepsilon_{\sigma }$ denotes the spin-dependent energy level of the QD, which can be controlled by modulating the gate voltage $V_{g}$ and λ is the coupling constant between the QD electron and photon mode, which is time-independent, and is different from the classical treatment that introduces a time-dependent oscillating energy level in QD [19,20]. The symbol *e* is the amount of charge on an electron, while that of $d_{\sigma }^{†}(d_{\sigma })$ is the corresponding creation (annihilation) operator of an electron in the QD, and U is the electron Coulomb correlation parameter. The last term describes the conduction electron hopping between the QD and electrode leads,

(3) $$H_{T}=\sum_{k\sigma ,\alpha \in L,R}T_{k\sigma ,\alpha }c_{k\sigma ,\alpha }^{†}d_{\sigma }^{}+H.c.$$

where $T_{k\sigma ,\alpha }$ is the spin-dependent tunneling co-efficient.

Using the Keldysh nonequilibrium Green’s function formalism, the current through the system can be obtained as [16,24,25,26],

(4) I=−1πeℏ∫dε {(fL−fR)∑σ=↑,↓ΓL,σΓR,σΓL,σ+ΓR,σImGσr(ε)}

where, *f_L_* and *f_R_* are the Fermi distribution functions for the left and right leads respectively, having different chemical potentials upon a voltage bias *μ_L_* − *μ_R_* = *eV_bias_*. This *V_bias_* is the bias voltage between the two electrode leads, while ℏ is Planck’s constant. *Γ_α ,σ_* represents the spin-dependent tunneling rates between QD and electrode leads, characterized by $\Gamma_{\alpha ,\sigma }=2\pi \underset{k}{\sum }{\left|T_{k\sigma ,\;\alpha }\right|}^{2}\delta \left(\hbar \omega -\varepsilon_{k\sigma ,\alpha }\right)$. $G^{r}$($G^{a}$) is the retarded (advanced) Green’s function for the QD electron coupled to the photon as well as to the leads, and $G^{<}$ is the lesser Green’s function. Equation [4] is only valid if the left and right tunneling rates are proportional to each other, i.e., $\Gamma_{L,\sigma }=\chi \Gamma_{R,\sigma }$, where χ is a constant [26].

It is convenient to eliminate the electron–photon coupling terms in the Hamiltonian by using a canonical transformation, i.e., $\tilde{H}=e^{s}He^{-s}$ with $s=\zeta (a^{†}-a)d_{\sigma }^{†}d_{\sigma }$ and $\zeta =\lambda /(\hbar \omega_{0})$ [16,24,25]. The transformed Hamiltonian becomes $\tilde{H}={\tilde{H}}_{el}+{\tilde{H}}_{ph}$, where ${\tilde{H}}_{\mathrm{ph}}=\hbar \omega_{0}a^{†}a$, and

(5) $$\begin{array}{cc} {\tilde{H}}_{el} & =\sum_{k\sigma ,\alpha \in L,R}\varepsilon_{k\sigma ,\alpha }c_{k\sigma ,\alpha }^{†}c_{k\sigma ,\alpha }^{}+\sum_{\sigma }{\tilde{\varepsilon }}_{{}_{\sigma }}d_{\sigma }^{†}d_{\sigma }^{}+\tilde{U}d_{↑}^{†}d_{↑}d_{↓}^{†}d_{↓}\\ & +\sum_{k\sigma ,\alpha \in L,R}\left({\tilde{T}}_{{}_{k\sigma ,\alpha }}c_{k\sigma ,\alpha }^{†}d_{\sigma }^{}+H.c.\right), \end{array}$$

where ${\tilde{\varepsilon }}_{\sigma }=\varepsilon_{\sigma }-\delta +eV_{g}$, $\tilde{U}=U-2\delta$, $\delta =g\hbar \omega_{0}$, $g=\zeta^{2}$ and ${\tilde{T}}_{k\sigma ,\alpha }=XT_{k\sigma ,\alpha }$ with $X=\exp\left[-\left(a^{†}-a\right)\right]$. ${\tilde{T}}_{k\sigma ,\alpha }$ can be approximated as its expectation value ${\tilde{T}}_{k\sigma ,\alpha }=\langle X\rangle T_{k\sigma ,\alpha }$, which is valid only when the hopping between QD and leads is small compared to the electron-photon interaction [16,24,25]. In the new representation, the retarded Green function of the system can be written as [25]:

(6) $$\begin{array}{cc} G^{r}(t) & =-i\theta (t)\langle e^{S}{\left[d_{\sigma }(t),d_{\sigma }^{†}\right]}_{+}e^{-S}\rangle \\ & ={\tilde{G}}^{r}(t){\langle X(t)X^{†}\rangle }_{ph}+\theta (t){\tilde{G}}^{<}(t)\left[{\langle X(t)X^{†}\rangle }_{ph}-{\langle X^{†}X(t)\rangle }_{ph}\right] \end{array}$$

By the equation of motion approach [26], under the infinite-U limit, it is easy to find the Fourier transform of ${\tilde{G}}_{\sigma }^{r}\left(t\right)$ as [16,25,26],

(7) $${\tilde{G}}_{\sigma }^{r\left(a\right)}\left(\omega \right)=\frac{1-\langle n_{\bar{\sigma }}\rangle }{\hbar \omega -{\tilde{\varepsilon }}_{\sigma }-{\tilde{\Sigma }}_{0\sigma }^{r\left(a\right)}\left(\hbar \omega \right)-{\tilde{\Sigma }}_{1\sigma }^{r\left(a\right)}\left(\hbar \omega \right)}$$

where the self-energy terms are [27,28]:

(8) $$\begin{array}{cc} {\tilde{\Sigma }}_{0\sigma }^{r(a)}(\hbar \omega ) & =\sum_{k,\alpha \in L,R}\frac{{\left|{\tilde{T}}_{k\sigma ,\alpha }\right|}^{2}}{\hbar \omega -\varepsilon_{k\sigma ,\alpha }\pm i0^{+}}={\left|\langle X\rangle \right|}^{2}\sum_{k,\alpha \in L,R}\frac{{\left|T_{k\sigma ,\alpha }\right|}^{2}}{\hbar \omega -\varepsilon_{k\sigma ,\alpha }\pm i0^{+}}\\ & =\sum_{\alpha \in L,R}\left\{PI\int_{-W}^{W}\frac{{\left|\langle X\rangle \right|}^{2}{\left|T_{\sigma ,\alpha }\right|}^{2}\rho (\varepsilon^{,})d\varepsilon^{,}}{\hbar \omega -\varepsilon^{,}}\mp i{\left|\langle X\rangle \right|}^{2}\sum_{k}{\left|T_{k\sigma ,\alpha }\right|}^{2}\pi \delta (\hbar \omega -\varepsilon_{k\sigma ,\alpha })\right\}\\ & =\sum_{\alpha \in L,R}\left\{PI\int_{-W}^{W}\frac{{\left|\langle X\rangle \right|}^{2}{\left|T_{\sigma ,\alpha }\right|}^{2}\rho (\varepsilon^{,})d\varepsilon^{,}}{\hbar \omega -\varepsilon^{,}}\mp i\frac{{\left|\langle X\rangle \right|}^{2}\Gamma_{\sigma ,\alpha }}{2}\right\} \end{array}$$

(9) $$\begin{array}{cc} {\tilde{\Sigma }}_{1\sigma }^{r\left(a\right)}\left(\hbar \omega \right) & =\underset{k,\;\alpha \in L,R}{\sum }\frac{{\left|{\tilde{T}}_{k\bar{\sigma },\alpha }\right|}^{2}f\left(\varepsilon_{k\bar{\sigma },\alpha }\right)}{\hbar \omega +{\tilde{\varepsilon }}_{\bar{\sigma }}-{\tilde{\varepsilon }}_{\sigma }-\varepsilon_{k\bar{\sigma },\alpha }\pm i0^{+}}\;\\ & ={\left|\langle X\rangle \right|}^{2}\sum_{\alpha \in L,R}\frac{\Gamma_{\alpha ,\bar{\sigma }}}{2\pi }\left[PI\int_{-W}^{W}\frac{f\left(\varepsilon \right)d\varepsilon^{\prime }}{\hbar \omega +{\tilde{\varepsilon }}_{\bar{\sigma }}-{\tilde{\varepsilon }}_{\sigma }-\varepsilon^{\prime }}\mp i\pi f_{\alpha }\left(\hbar \omega +{\tilde{\varepsilon }}_{\bar{\sigma }}-{\tilde{\varepsilon }}_{\sigma }\right)\right]\\ & =-{\left|\langle X\rangle \right|}^{2}\sum_{\alpha \in L,R}\frac{\Gamma_{\alpha ,\bar{\sigma }}}{2\pi }\left\{\pm i\pi f_{\alpha }\left(\hbar \omega +{\tilde{\varepsilon }}_{\bar{\sigma }}-{\tilde{\varepsilon }}_{\sigma }\right)+ln\frac{2\pi k_{B}T}{W}+\Psi \left(\frac{1}{2}\mp i\frac{\hbar \omega +{\tilde{\varepsilon }}_{\bar{\sigma }}-{\tilde{\varepsilon }}_{\sigma }-\mu_{\alpha }}{2\pi k_{B}T}\right)\right\} \end{array}$$

where *PI* represents principal value integral, Ψ is the digamma function, and *W* is the conduction half-bandwidth of the electrode lead. ${\tilde{\Sigma }}_{0\sigma }^{r}$ is the self-energy due to the tunneling into the leads without the influence of electron-electron interaction in QD, while ${\tilde{\Sigma }}_{1\sigma }^{r}$ is the modification to the self-energy ${\tilde{\Sigma }}_{0\sigma }^{r}$ because of the electron-electron interaction in QD [26]. For convenience, we consider a constant conduction density of states ρ(ε)=1/2W for −W<ε<W, and $T_{k\sigma ,\;\alpha }=T_{\sigma ,\;\alpha }$ which is independent of wave vector *k* [28].

The situations for thermal and Fock state optical fields are formulated as follows.

A. If the optical field is in the thermal state $\rho_{thermal}=\sum_{N=0}^{\infty }\frac{N_{th}^{N}}{{\left(1+N_{th}\right)}^{N+1}}|N\rangle \langle N|$. Although the results are similar to those reported in [25] of the phonon-assisted inelastic transport through a single QD with ferromagnetic electrodes for the thermal state optical field, it was mainly applied to perform a contrastive analysis for the case of the Fock state. Furthermore, the phonon-assisted spin-polarized tunneling through a QD, interacting with the thermal state phonon field under a finite Coulomb correlation parameter, has been studied in [16]. The average photon number of the thermal state is assumed to be an independently varied parameter in this paper, because the temperature of the thermal optical field can be controlled differently from the temperature of the electrode leads.

B. If the optical field is in the Fock state $|N_{Fock}\rangle$, using some algebra, one can obtain $\langle X^{†}X\left(t\right)\rangle_{Fock}=\langle X\left(t\right)X^{†}\rangle_{Fock}^{*}$ (see Appendix A)

(10) $$\langle X\left(t\right)X^{†}\rangle_{Fock}=e^{-g}\sum_{k=0}^{\infty }\sum_{l=0}^{N_{Fock}}\frac{g^{l}{\left(-1\right)}^{k}N_{Fock}!}{{\left(l!\right)}^{2}\left(N_{Fock}-l\right)!}L_{k}^{2l-k}\left(g\right)e^{-i\left(k-l\right)\omega_{0}t}$$

where $L_{k}^{2l-k}$ is Laguerre polynomials, $N_{Fock}$ is the photon number in the Fock state. Thus, the corresponding imaginary part of $G_{\sigma }^{r}\left(\omega \right)$ reads (see Appendix B)

(11) ImGσr=−(1−〈nσ¯〉)∑k=0∞∑l=0NFockΦkl{[1−f¯σ(ε−(k−l)ℏω0)][Γ˜σ−Aσ(ε−(k−l)ℏω0)][ε−ε˜σ−(k−l)ℏω0−Bσ(ε−(k−l)ℏω0)]2+[Γ˜σ−Aσ(ε−(k−l)ℏω0)]2+f¯σ(ε+(k−l)ℏω0)[Γ˜σ−Aσ(ε+(k−l)ℏω0)][ε−ε˜σ+(k−l)ℏω0−Bσ(ε+(k−l)ℏω0)]2+[Γ˜σ−Aσ(ε+(k−l)ℏω0)]2}

where

(12) $$\Phi_{kl}=e^{-g}\frac{N_{Fock}!\;g^{l}{\left(-1\right)}^{k}}{{\left(l!\right)}^{2}\left(N_{Fock}-l\right)!}L_{k}^{2l-k}\left(g\right)$$

$A_{\sigma }$ and $B_{\sigma }$ are the imaginary part and the real part of ${\tilde{\Sigma }}_{1\sigma }^{r}\left(\omega \right)$, respectively. ${\tilde{\Gamma }}_{\sigma }=\left({\tilde{\Gamma }}_{L,\sigma }\;+{\tilde{\Gamma }}_{R,\sigma }\right)/2\approx i{\tilde{\Sigma }}_{0\sigma }^{r}$, ${\tilde{\Gamma }}_{\alpha ,\sigma }=\Gamma_{\alpha ,}{\left|\langle X\rangle \right|}^{2}$. ε=ℏω. f¯σ(ε)=[ΓL,σfL(ε)+ΓR,σfR,σ(ε)](ΓL,σ+ΓR,σ).

The averaged occupation number $\langle n_{\sigma }\rangle$ of the spin σ electron in a QD can be calculated by the self-consistent equations as follows [26]:

(13) 〈nσ〉=−∫dεπf¯σ(ε)ImGσr(ε)

This equation is obtained from the fluctuation–dissipation theorem [26].

## 3. Discussion

We first discuss the influences of the thermal and Fock state optical fields on the coupling coefficient between a QD and ferromagnetic electronic leads after the canonical transformation.

The effective QD–lead coupling coefficient ${\tilde{T}}_{k\sigma ,\alpha }$ may be approximated as its expectation value ${\tilde{T}}_{k\sigma ,\alpha }=\langle X\rangle T_{k\sigma ,\alpha }$ after the canonical transformation [24,25]. In the new representation, the electrons are dressed with a photon cloud with the interaction of QD and leads. It is easy to find ${\langle X\rangle }_{thermal}=\exp\left[-g\left(N_{th}+1/2\right)\right]$ for the thermal state and 〈X〉Fock=exp(−g2)LNFock(g) for the Fock state (see Appendix C).

**Figure 2:**
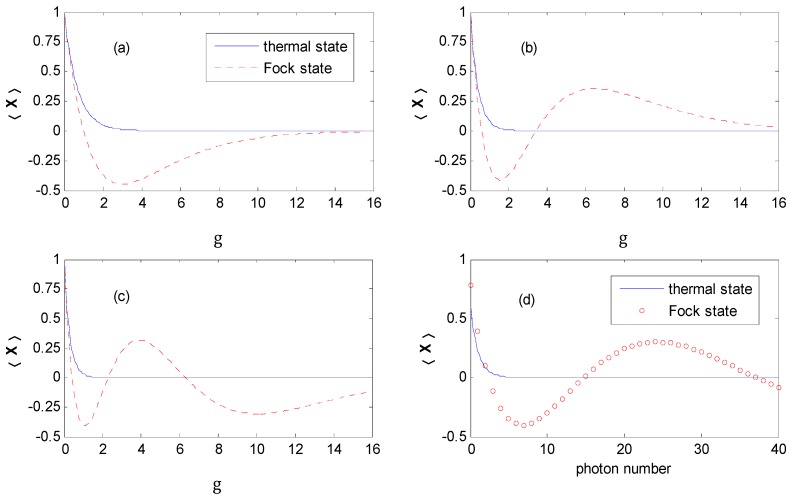
〈X〉 as a function of *g* for the thermal and Fock state optical fields. (**a**) *N_th_ = N_Fock_* = 1. (**b**) *N_th_ = N_Fock_* = 2. (**c**) *N_th_ = N_Fock_* = 3. (**d**) 〈X〉 as a function of photon number for the thermal and Fock state optical fields, and *g* = 0.5.

Figure 2a shows that 〈X〉 decreases exponentially to zero with the increase of *g* for the thermal state, while it reduces to a certain negative value, and then tends to zero gradually with increasing $g={\left(\lambda /\hbar \omega_{0}\right)}^{2}$ for the Fock state. Figure 2b,c show that 〈X〉 will decrease and oscillate around the abscissa axis when approaching zero for the Fock state with an increasing photon number.

For the case of the Fock state $|N_{Fock}\rangle$, the curve crosses the abscissa axis $N_{Fock}$ times, for example, once for |1〉, twice for |2〉, and three times for |3〉 and so on. The nonmonotonicity of Laguerre functions results in the oscillatory behavior. For effective coupling coefficients ${\tilde{T}}_{k\sigma ,\alpha }$, the real zeros reveal that the tunneling can be critically suppressed at certain values of the *g* for the Fock state. We can also find that the ${\tilde{T}}_{k\sigma ,\alpha }$ may possess a negative value at a certain regime of *g*. It reveals that the Fock state optical field would change phase for ${\tilde{T}}_{k\sigma ,\alpha }$ when QD electron dressing with a photon cloud interacts with the electronic leads. This attests to the existence of quantum interference between transmissions through different photon sideband channels [29].

Then, we analyzed the influences of the thermal and Fock state optical fields on the effective tunneling rate ${\tilde{\Gamma }}_{\alpha ,\sigma }$ of a QD and the ferromagnetic electronic leads after the canonical transformation. The expression of the effective coupling rate is ${\tilde{\Gamma }}_{\alpha ,\sigma }=\Gamma_{\alpha 0}{\left|\langle X\rangle \right|}^{2}$. Figure 2d shows 〈X〉 as a function of the photon number for the thermal and Fock state optical fields.

From Figure 2, we can find that the effective coupling rate ${\tilde{\Gamma }}_{\alpha ,\sigma }$ is suppressed by a factor ${\left|\langle X\rangle \right|}^{2}$, which is determined by the parameter *g* and the photon number of the optical field. The real zeros appear at a certain value of *g* for the Fock state.

From Equation (8), Equation (9) and Equation (11), we can find that optical fields impact the shift of photon sidebands by the product factor ${\left|\langle X\rangle \right|}^{2}$ in $B_{\sigma }$, and that they affect the line width of photon sidebands with the product factor ${\left|\langle X\rangle \right|}^{2}$ in $A_{\sigma }$ and ${\tilde{\Gamma }}_{\sigma }$, respectively. The amplitudes of photon sidebands are influenced by optical fields using the coefficients $\langle X^{†}X\left(t\right)\rangle$ and $\langle X\left(t\right)X^{†}\rangle$ in Equation (6), and $\langle n_{\bar{\sigma }}\rangle$ in Equation (7) which is determined by the self-consistent Equation (13).

${|\langle X|\rangle }^{2}$ not only depends on the electron–photon coupling strength, but also on the photon intensity for the quantum state optical field. Figure 2 shows that ${\left|{\langle X\rangle }_{th}\right|}^{2}$ decreases monotonously with the increase in the electron–photon coupling strength or averaged photon number for the thermal state optical field, while it becomes oscillatory upon an increasing electron–photon coupling strength or photon number for the Fock state case.

### 3.1. Density of State (DOS)

Here, we investigate the properties of *DOS* in a QD under different parameters. In the following discussions, we set $\varepsilon_{\sigma }=\varepsilon_{\bar{\sigma }}=E_{0}=E_{F}+eV_{g}-\delta$ with $E_{F}=0$, which is the equilibrium chemical potential of the left (right) electrode lead in the absence of gate voltage $V_{g}$. $\mu_{L}$($\mu_{R}$) is the chemical potential of the left (right) electrode, which is taken as $\mu_{L\left(R\right)}=E_{F}\pm eV_{bias}/2$. We also introduce the tunneling rate between QD and lead $\Gamma_{\alpha }$ as $\Gamma_{L}=\Gamma_{0}\left(1+P\sigma_{Z}\right)$ and $\Gamma_{R}=\Gamma_{0}\left(1\pm P\sigma_{Z}\right)$ with +(−) for the *P* and *AP* magnetization alignment of the ferromagnetic electrodes, where $\Gamma_{0}$ describes the tunneling rate between the QD and lead without internal magnetization. *P* is the spin polarization of the ferromagnetic electrodes. $\beta =1/k_{B}T_{leads}$, $T_{leads}$ is the temperature of the electrode leads. For simplicity, we state that the energy unit is taken as the photon energy $\hbar \omega_{0}$, and the unit of β is $1/\hbar \omega_{0}$. *W* is set as $100\hbar \omega_{0}$ in this article.

Moreover, when the temperature of the electrode lead approaches the Kondo temperature $T_{k}$, the decoupling approximation, used in the derivation of Equation (7), could lead to some drawbacks due to the logarithmic divergence of the digamma function. Specifically, the divergence of the digamma function could lead to incorrect behavior of the *DOS* at the Fermi level [30]. Thus, in this paper, we assume that the temperature of the electrode lead $T_{leads}$ is higher than the Kondo temperature $T_{k}$ determined by the method in [31].

Up to now, as far as we know, there is no experiment on NDC based on a photon-assisted QD system. However, there exist several experiments on NDC based on a phonon-assisted carbon tube system [32,33]. The NDC features can be described quite simply using a generic model which consists of a quantum dot with a single-orbital level, coupled to an on-site single phonon mode, and connected to leads by tunnel junctions [15]. Thus, we set the numeric values of the parameters in this paper with reference to that in [15,24,25,32,33]. We also set the photon energy $\hbar \omega_{0}$ as an elementary scale, and give the units of other parameters with $\hbar \omega_{0}$ and the amount of charge on an electron *e*.

Here, we begin to discuss *DOS* for the system under different parameters.

**Figure 3:**
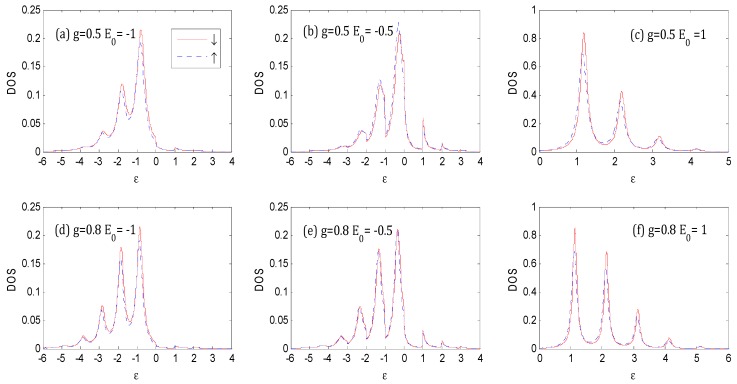
Density of states (*DOS*) for the vacuum state at *P* = 0.1, $\Gamma_{0}=0.2\hbar \omega_{0}$, *V_bias_* = 0 and β=100 in the *P* configuration. The unit of ε and *E*_0_ is $\hbar \omega_{0}$.

Because the thermal state and Fock state become the vacuum state when the photon number equals to zero, Figure 3 shows the *DOS* changes with *E*_0_ and *g* for the vacuum state_._ One can find that the main peaks appear near the location at $\varepsilon =E_{0}$, which are the channels for electron transport without any participation of a photon. Figure 3b,e show that both sides of the main peak appear as a series of satellite peaks. The electron–photon interaction has at least two effects: It results in an energy shift δ of the elastic peak position relative to that for *g* = 0, and leads to a set of new peaks induced by photon emission. The *n*th sideband peak on the right (left) side with respect to the main peak near the location at *E*_0_ corresponds to the *n*th photon emission sideband of the electron (hole) transport. The electron (hole) contribution comes from the first (second) term on the right-hand side of Equation (11), respectively [16,24,25].

Compared with that of *E*_0_ = −1$\hbar \omega_{0}$, the main peak and sideband width are compressed, the peaks of sidebands become larger and evidently shift to the right with the increase in *E*_0_, as shown in Figure 3a–f. The weight of photon sidebands transfers gradually from the left to the right of the main peak, and the line shape of each photon satellite shows a jump discontinuity [34] with respect to ω in Figure 3b,e [24]. This is because when the effective QD electron energy level approaches to Fermi surface, the electrons (holes) near the Fermi surface dominate the tunneling process, and the sharp change of Fermi functions near the Fermi surface level at the lower temperature results in the jump discontinuity. This behavior is consistent with that obtained for electron–phonon coupling in [16,24,25]. An increasing number of photon sidebands appear with increasing *g*, and all peak values of the sidebands increase as *g* becomes greater, even in the vacuum state of the optical field, which is quite different from those of the case under classical treatment for the external field [20]. The width of main peak is compressed as the increase of *g.*

**Figure 4:**
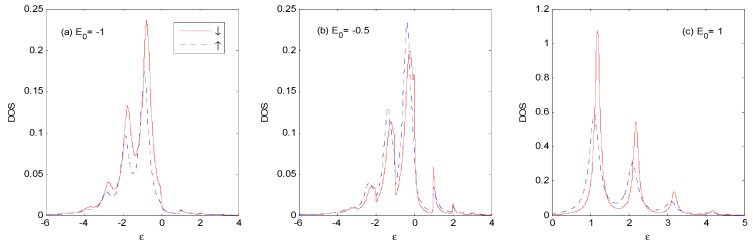
Density of states (*DOS*) as a function of ε for the vacuum state. The parameters for (**a**), (**b**) and (**c**) are the same as that of Figure 3a–c except for *P* = 0.31, respectively. The unit of ε and *E*_0_ is $\hbar \omega_{0}$.

Comparing Figure 4a–c with Figure 3a–c respectively, although the difference between spin up *DOS* and spin down *DOS* is larger as *P = 0.31* [25] and the higher spin polarization could result in a higher spin-dependent splitting in *DOS*, the envelopes of *DOS* are similar to each other respectively. Without loss of generality, we set *P* = 0.1 throughout this paper.

**Figure 5:**
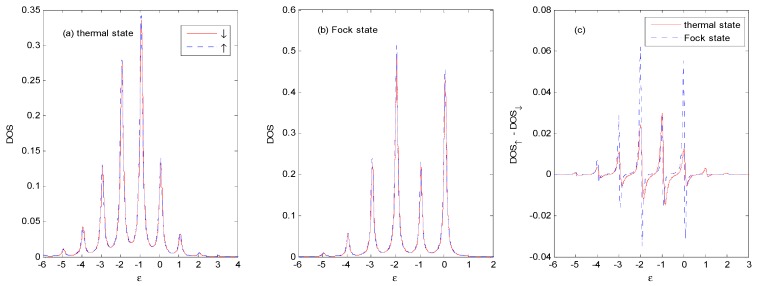
*DOS* for the thermal and Fock state optical fields at *E*_0_ = −1$\hbar \omega_{0}$, *g* = 0.5, *P* = 0.1, $\Gamma_{0}=0.2\hbar \omega_{0}$, *V_bias_* = 0, *β* =100 and *N_th_* = *N_Fock_* = 1 in the *P* configuration. The unit of ε is $\hbar \omega_{0}$.

Figure 5 shows the different stages of evolution of the *DOS* with the thermal and Fock state optical fields for *E*_0_ = −1$\hbar \omega_{0}$ and *N_th_* = *N_Fock_* = 1. Compared with Figure 3a, when the photon intensity is non-zero, the weight of main peak near $\varepsilon =-1\hbar \omega_{0}$ becomes larger, accompanied with compressed width, and photon satellites are evident on both sides of the main peak. Photon satellites exist above the Fermi surface, while they are almost non-existent for the vacuum state in Figure 3a, as it can only absorb photons from the external optical field.

As there is definitely one photon in the Fock state |1〉 (while the one-photon probability is only 25%, and the zero-photon probability is 50% in the thermal state, with an average photon number of *N_th_* = 1), the photon sideband peaks for the case of the Fock state are higher, and the weight of the main peak is lower than those of the thermal state. There are photon satellites above the Fermi surface for the case of the thermal state because of the non-vanishing multi-photon probabilities.

Figure 5c shows that the *DOS* is spin-dependent because of the spin polarization of the ferromagnetic electrodes [25]. There is a small spin splitting of the sideband in the *P* configuration, which comes from the spin-dependent real part of the self-energy in Equation (9). The spin splitting of *DOS* increases with the increasing value of the spin polarization parameter *P* of the ferromagnetic electrodes.

**Figure 6:**
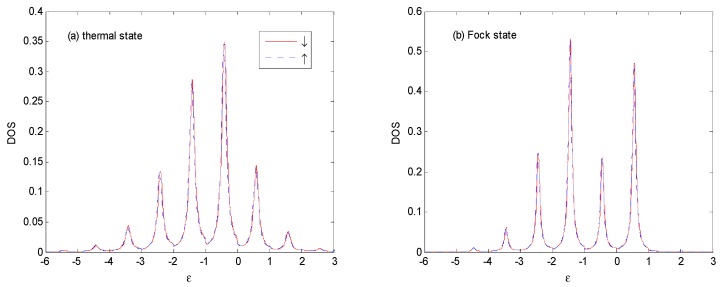
*DOS* for the thermal and Fock state optical fields with the same parameters as those in Figure 5, except that *E*_0_ = −0.5$\hbar \omega_{0}$. The unit of ε is $\hbar \omega_{0}$.

When *E*_0_ changes to −0.5$\hbar \omega_{0}$ in Figure 6, the *DOS* is obviously changed in the thermal and Fock state optical fields. The weight and width of main peak is almost the same as that in Figure 5. The weight of the photon sidebands transfers gradually from the left to the right of the main peak, such as those in Figure 5. There are photon satellites above ε *=* 1$\hbar \omega_{0}$ for the thermal state because the multi-photon probabilities are non-vanishing.

**Figure 7:**
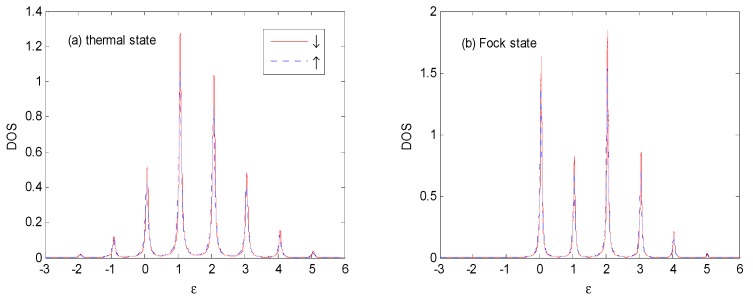
*DOS* for the thermal and Fock state optical fields with the same parameters as those in Figure 5, except for *E*_0_ = 1$\hbar \omega_{0}$. The unit of ε is $\hbar \omega_{0}$.

From Figure 5 to Figure 7, one can find that the *DOS* noticeably changed with the increasing of *E*_0_ in the thermal and Fock state optical fields. The weight of main peak near $\varepsilon =1\hbar \omega_{0}$ becomes larger accompanied by a compressed width for thermal state, and the weight of main peak becomes smaller, accompanied by a compressed width for Fock state. Almost all of the photon satellites are transferred to the right side of the main peak for the Fock state because pure electron transport predominantly contributes to the *DOS* [25]. There are photon-satellites that exist below the Fermi surface for the thermal state because the multi-photon probabilities are non-vanishing, while this is not the case for the Fock state optical field.

**Figure 8:**
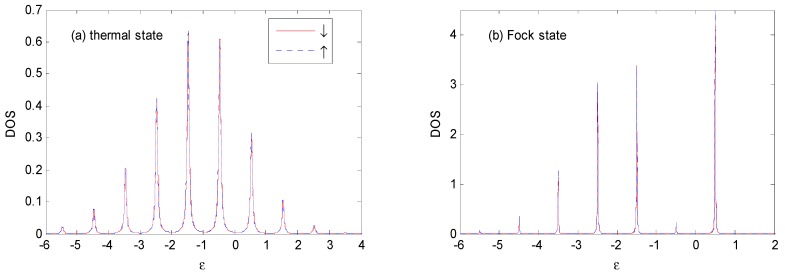
*DOS* for the thermal and Fock state optical fields with the same parameters as those in Figure 6, except that *g* = 0.8. The unit of ε is $\hbar \omega_{0}$.

Comparing Figure 8 with Figure 6, we find that the *DOS* for different states evidently varies with the parameter, *g*. With the increase of the electron–photon coupling parameter, *g*, the height of the main peak decreases, while the heights of the photon-assisted sideband peaks increase, which indicates a higher probability of photon–emission or photon absorption. The widths of these peaks become narrower since, according to the discussions of Figure 2, the system is trapped in a region of exponentially-suppressed tunneling rates with increasing values of the *g* parameter [16]. We can also find that the pedestals of each sideband overlap for smaller electron–photon coupling parameters, *g,* while they tend to have well-defined boundaries at larger electron–photon coupling parameters, *g*. With the increase of *g*, an increasing number of photon sidebands appear, and the peak value increases.

**Figure 9:**
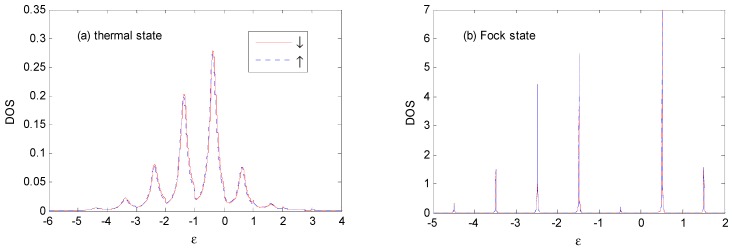
*DOS* for the thermal and Fock state optical fields with the same parameters as those in Figure 6, except that in (**a**) $\langle N_{th}\rangle =0.6$ and in (**b**) *N_Fock_* = 2. The unit of ε is $\hbar \omega_{0}$.

By the comparison of Figure 9 with Figure 3b, Figure 3e and Figure 6, it could be found that the amplitudes of the main peak and photon satellites increase with increasing photon intensity. Comparing Figure 9b with Figure 6b, one can find that the weight of the main peak near $\varepsilon \approx -0.5\hbar \omega_{0}$ is almost equal to zero and a new sideband appears at $\varepsilon \approx 1.5\hbar \omega_{0}$ when the photon number of the Fock state equals 2. The reason is that the source lead electron (hole) at the Fermi surface can absorb (emit) 2 photons to get to $\varepsilon \approx 1.5\hbar \omega_{0}$ and form a new sideband. It is evident that the weights of photon satellites increase, while the peak widths become narrower, when the photon number increases from 1 to 2. The reason for this is that the suppression factor of the tunneling rate is enhanced as the photon number increases, which is discussed in Figure 2.

**Figure 10:**
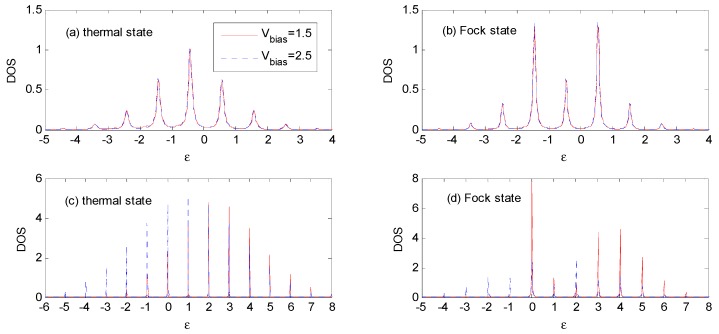
Total *DOS* (the sum of spin up and down *DOS*) for the thermal and Fock state optical fields with the same parameters as those in Figure 6, except that *E*_0_ = −0.5$\hbar \omega_{0}$ and *g* = 0.5 for (**a**) and (**b**); *E*_0_ = 1.0$\hbar \omega_{0}$ and *g* = 1.5 for (**c**) and (**d**). The unit of ε is $\hbar \omega_{0}$ and the unit of V_bias_ is $\hbar \omega_{0}/e$.

Total *DOS* (the sum of spin up and down *DOS*) are shown in Figure 10 at different bias voltage in the thermal and Fock state optical fields with different effective QD energy levels and different electron–photon coupling constants. It can be seen that there are slight differences in the total *DOS* for different bias voltages at *E*_0_ = −0.5$\hbar \omega_{0}$ and *g* = 0.5; however, the height and location of the sideband is quite different for diverse bias voltage at *E*_0_ = 1$\hbar \omega_{0}$ and *g* = 1.5. There are new sidebands that appear at $\approx -3,-4,-5\hbar \omega_{0}$ for *V_bias_* = 2.5 (blue line) with respect to *V_bias_* = 1.5 (red line) in Figure 10c, and new sidebands appear at $\approx -1,-2,-3\hbar \omega_{0}$ for *V_bias_* = 2.5 with respect to *V_bias_* = 1.5 in Figure 10d. The weights of the sidebands that appear at $\approx -2,\;-1,\;0,\;1\hbar \omega_{0}$ for *V_bias_* = 2.5 are different from that for *V_bias_* = 1.5 in Figure 10c respectively, and the weights of sidebands appearing at $\approx 0,2,\;3,\;4,\;5\hbar \omega_{0}$ for *V_bias_* = 2.5 are quite different from that for *V_bias_* = 1.5 in Figure 10d respectively. This can lead to complex current–voltage characteristics in the thermal and Fock states, which are discussed in the next section.

**Figure 11:**
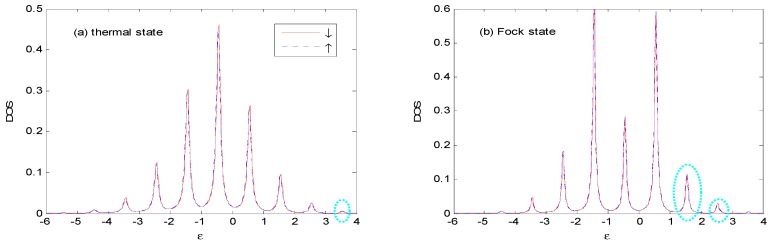
*DOS* for the thermal and Fock state optical fields with the same parameters as those in Figure 6, except that *β* =1. The unit of ε is $\hbar \omega_{0}$.

Figure 11 demonstrates *DOS* at different leads’ temperatures for the thermal and Fock state optical fields. An increasing number of photon emission (absorption) sidebands appear (within circle) with the increase of leads’ temperature, and the peak value of the sideband increases with the increasing of leads’ temperature because this results in an increasing number of leads’ electrons above the Fermi surface. With the increasing of leads’ temperature, the weight of main peak becomes larger, accompanied with compressed width for both thermal state and Fock state compared with Figure 6.

**Figure 12:**
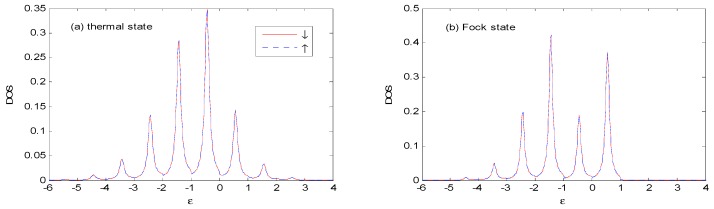
*DOS* for the thermal and Fock state optical fields with the same parameters as those in Figure 6, except that the *AP* configuration is used. The unit of ε is $\hbar \omega_{0}$.

Comparing Figure 12 with Figure 6, it can be seen that the properties of the *DOS*, for the antiparallel (*AP*) configuration are similar to those in parallel (*P*) configuration, and there is no difference in *DOS* between the spin-down and spin-up orientations at zero bias voltage. Thus, it is not necessary to discuss these further for the *AP* configuration.

### 3.2. Current

From the discussions above, we find that the *DOS* is sensitive to the QD energy level, electron–photon interaction and bias voltage at the leads, and the evolution characteristics of *DOS* for the cases of external optical fields in the thermal and Fock states are quite different. Thus, in this section, we will investigate the properties of the tunneling current–voltage characteristics upon changing the QD energy level, and the electron–photon interaction for the cases of external optical fields in the thermal and Fock states.

Because the mismatch of spin-dependent Fermi wave vectors for the *AP* configuration can result in a decrease of the transmission probability of electrons or holes, the current for the *P* magnetization alignment is significantly larger than that for the *AP* configuration [25]. Therefore, we mainly discuss the tunneling current for the *P* configuration here. In the following discussion, we denote $I_{↑}-I_{↓}$ as $I_{s}$, $I_{↑}+I_{↓}$ as *I*, and the differential conductance $G=dI/dV_{bias}$, respectively.

**Figure 13:**
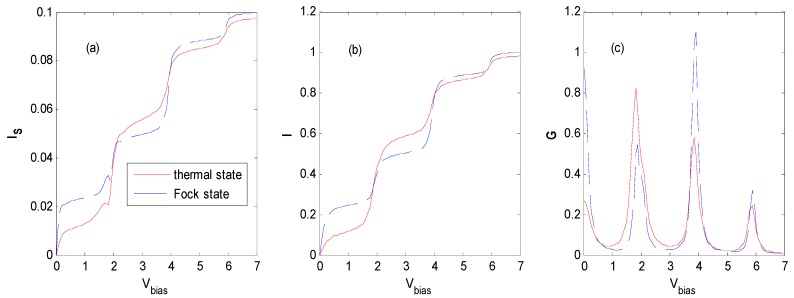
Tunneling current and differential conductance for the thermal and Fock state optical fields at *E*_0_ = −1$\hbar \omega_{0}$, *g* = 0.5, *P* = 0.1, $\Gamma_{0}=0.2\hbar \omega_{0}$, *β* =100, *N_th_* = *N_Fock_* = 1 in the *P* configuration. The unit of current is $\frac{e}{\pi \hbar }\Gamma_{0}$, the unit of V_bias_ is $\hbar \omega_{0}/e$ and the unit of differential conductance is $\frac{e^{2}}{5\pi \hbar }$.

Figure 13 demonstrates the well-known staircase shape current for the different states of optical fields [16,20,24,25]. Because the QD electron has a non-vanishing probability of occupying the photon sidebands, once photon sidebands enter the bias window one by one, the photon-assisted channel would open. Thus, the tunneling processes may be mediated by the photon energy levels, which results in additional steps in the current. It shows that the currents for the cases of the thermal and Fock states tend to have a similar value of saturation at a higher bias voltage, while the currents evolve differently at lower bias voltage. From Figure 13, it can be found that the currents tend to reach their saturation value in four steps in the thermal and Fock states upon increasing the bias voltage, even when there is a little difference in the current–voltage characteristics between them. The different tunneling properties of a QD electron dressed with different-state photons result in a different shape of the current curve.

Figure 13a also demonstrates that there is a small fluctuation of spin current at each step because of the tiny difference of spin *DOS* discussed in Figure 5c.

By comparison of Figure 13c and Figure 5, it can be found that differential conductance reflects the *DOS* properties of a QD system. As the increase of bias voltage, the differential conductance peaks appear only when the lead chemical potential is aligned with the peaks of the *DOS*. In the vicinity of the zero bias voltage regions, where the sequential tunneling current is exponentially suppressed, the differential conductance is due to higher-order tunneling processes. The amplitude of each differential conductance peak reveals the amplitude of the main peak and photon sideband of the *DOS* of a QD system.

As the effective energy level *E*_0_ changes to −0.5$\hbar \omega_{0}$ and 1$\hbar \omega_{0}$, respectively, they have similar current–voltage characteristics for spin current and total current in Figure 13, except that the fluctuation of the spin current at each step becomes larger as the effective energy level *E*_0_ changes from −1$\hbar \omega_{0}$ to −0.5$\hbar \omega_{0}$ and 1$\hbar \omega_{0}$, respectively.

**Figure 14:**
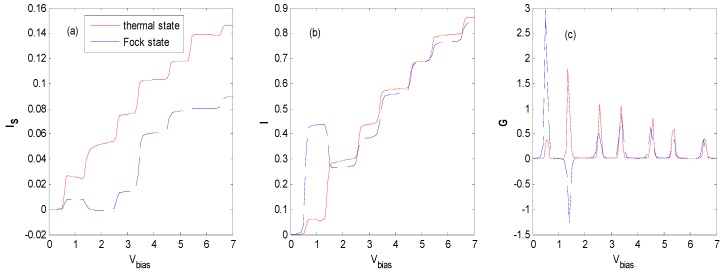
Tunneling current and differential conductance for the thermal and Fock state optical fields with the same parameters as Figure 13 except for *E*_0_ = 0.7$\hbar \omega_{0}$, *g* = 1.5. The unit of current is $\frac{e}{\pi \hbar }\Gamma_{0}$, the unit of V_bias_ is $\hbar \omega_{0}/e$ and the unit of differential conductance is $\frac{e^{2}}{5\pi \hbar }$.

**Figure 15:**
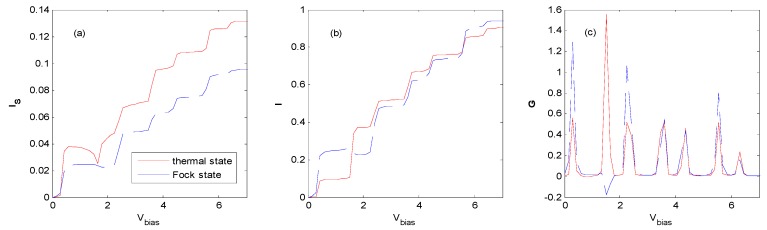
Tunneling current and differential conductance for the thermal and Fock state optical fields with the same parameters as Figure 14 except for *E*_0_ = 0.8$\hbar \omega_{0}$, *g* = 0.6, *N_th_* = *N_Fock_* = 3. The unit of current is $\frac{e}{\pi \hbar }\Gamma_{0}$, the unit of V_bias_ is $\hbar \omega_{0}/e$ and the unit of differential conductance is $\frac{e^{2}}{5\pi \hbar }$.

**Figure 16:**
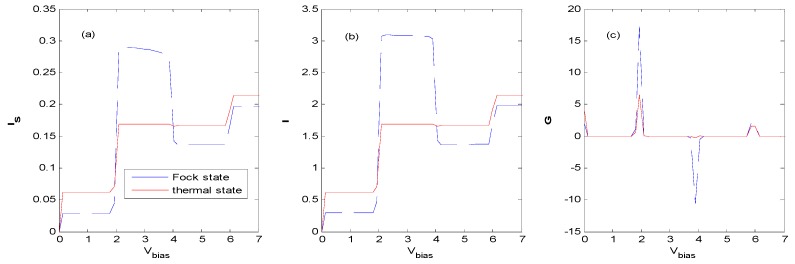
Tunneling current and differential conductance for the thermal and Fock state optical fields with the same parameters as Figure 14 except for *E*_0_ = 2$\hbar \omega_{0}$, *g* = 2.3, *N_th_* = *N_Fock_* = 3. The unit of current is $\frac{e}{\pi \hbar }\Gamma_{0}$, the unit of V_bias_ is $\hbar \omega_{0}/e$ and the unit of differential conductance is $\frac{e^{2}}{5\pi \hbar }$.

There are two types of competing mechanisms. One is that a stronger electron–photon interaction can enhance the photon emission (absorption), through which the photon-assisted tunneling channel forms, which is favorable to the formation of photon satellites. The other is an exponential suppression of the effective coupling between the QD and leads. This competition may result in NDC in the photon-assisted spin-polarized tunneling through an interacting QD with antiparallel configuration (AP) under a finite Coulomb correlation parameter [16]. From the discussions of Figure 2, one can find that the tunneling rates between the QD and leads can approach zero in some regions of the electron–photon interaction, which means the tunneling through QD is critically suppressed. Thus, we could investigate NDC in the vicinity of zero regions in Figure 2 and Figure 14, Figure 15 and Figure 16 confirms this assumption.

With the proper effective QD energy level, Figure 14, Figure 15 and Figure 16 show that there are NDCs for a QD system interacting with an external field of the Fock state at the vicinity of the first zero as the photon number is 1 (Figure 14), at the vicinity of the first zero (Figure 15) and the second zero (Figure 16) as the photon number is 3, respectively. NDC also appears in a similar parameter region as when the photon number is 2, or with the *AP* configuration (not shown). Figure 16 shows that a subtle NDC also exists for the case of the thermal state with the same parameter. This can be deduced from Figure 10, where the height and the location of the photon sideband peaks change as the bias voltage changes, indicating the change in the probability of photon-emission or photon-absorption. If the height of the photon sideband or the number of photon sidebands inside the bias voltage window decreases with the increasing bias voltage, NDC appears. The physical process can also be described as follows [16]. When electrons tunnel through the QD from the left electrode to the right one in the vicinity of the zero bias voltage regions, higher-order tunneling processes exist, while the sequential tunneling is exponentially suppressed. If the *V_bias_* is sufficiently large, the photon sideband enters the bias voltage window, and photon-assisted tunneling current starts to flow through the QD. Subsequently, the tunneling current plateau is observed. Above the current plateau, an NDC is observed because the tunneling current is suppressed by an electron residing on the QD. Finally, when the next photon sideband crosses the Fermi level of the source lead, the current increases again and shows a tendency towards saturation.

The Hamiltonian model discussed in this paper is similar to that of a single molecular junction in [16], except that the state of the external field is the Fock state in this paper. However, we find that obvious NDC appears in a single QD system assisted by the Fock state optical field with both *P* and *AP* configurations, while evident NDC only emerges in an *AP* configuration in [16]. In this paper, NDC can be obtained for a wide range of electron–photon interaction parameters at the vicinity of the zeros of the Laguerre function (see Figure 2), because there is an increasing number of zeros when the photon number of the Fock state increases.

## 4. Conclusions

Based on the effect of quantum treatment on an optical field, we have investigated the quantum transport through the single QD with ferromagnetic electrodes in the presence of an electron–photon interaction by the Keldysh nonequilibrium Green’s function approach. The important results can be briefly summarized as follows.

Firstly, owing to the interaction with optical fields, the shift of photon sidebands is dependent on the electron–photon coupling strength and the photon intensity. For an increasing electron–photon coupling strength, the shift is monotonically decreasing for the case of the thermal state optical field, while it is oscillatory for the Fock state optical field because of the nonmonotonicity of Laguerre functions.

Secondly, the *DOS* and the tunneling current are sensitive to the QD energy level adjusted by the gate voltage and the electron–photon interaction. The evolution properties of *DOS* and tunneling current for photon-assisted spin-polarized tunneling with the Fock state of optical field, are quite different from that with the thermal state case.

Thirdly, obvious NDC can be found in both *P* and *AP* configuration QD systems assisted by the Fock state optical field in a wide range of electron–photon interaction parameters, while evident NDC usually only arises in an *AP* configuration QD system assisted by the thermal state optical field.

These important results enlighten us that a single-electron tunneling transport in a QD can be actively manipulated and controlled by quantum states of the optical field. Meanwhile, the information of the spectral function of QD and the interaction with external optical fields could be inferred from the spectra of the tunneling current. Furthermore, NDC has potential applications in the realization of low-power memory devices and logic circuits. Thus, the results would deepen the understanding of single-electron tunneling transport properties in QD, enrich the control techniques for the transport of QD electrons, and open a new door for constructing nanostructured devices and spin-dependent devices.

## Appendix A. The Derivation of Equation (10) and Equation (12)

Using the same method of the appendix A of [35], one can obtain:

(A1) $$\langle N_{Fock}\left|e^{u^{*}a^{†}}e^{-ua}\right|N_{Fock}\rangle =\sum_{l=0}^{N_{Fock}}\frac{{\left(-1\right)}^{l}N_{Fock}!}{{\left(l!\right)}^{2}\left(N_{Fock}-l\right)!}{\left({\left|u\right|}^{2}\right)}^{l}=L_{N_{Fock}}\left({\left|u\right|}^{2}\right)$$

where $u=\zeta \left(1-e^{-i\omega_{0}t}\right)$, $L_{N_{Fock}}\left(x\right)$ is Laguerre Polynomial. Thus, the correlation function can be rewritten as:

(A2) $$\begin{array}{cc} \langle X\left(t\right)X^{†}\rangle_{Fock} & =exp\left(-\zeta^{2}\left(1-e^{-i\omega_{0}t}\right)\right)\langle N_{Fock}\left|\exp\left(\zeta (1-e^{i\omega_{0}t})a^{†}\right)exp\left(-\zeta (1-e^{-i\omega_{0}t})a\right)\right|N_{Fock}\rangle \\ & \;=exp\left(-\zeta^{2}\left(1-e^{-i\omega_{0}t}\right)\right)\sum_{l=0}^{N_{Fock}}\frac{{\left(-1\right)}^{l}N_{Fock}!}{{\left(l!\right)}^{2}\left(N_{Fock}-l\right)!}{\left({\left|u\right|}^{2}\right)}^{l}\\ & \;=e^{-\zeta^{2}}\sum_{l=0}^{N_{Fock}}\frac{{\left(-1\right)}^{l}N_{Fock}!}{{\left(l!\right)}^{2}\left(N_{Fock}-l\right)!}{\left({\left|u\right|}^{2}\right)}^{l}e^{\zeta^{2}e^{-i\omega_{0}t}}\\ & \;=e^{-\zeta^{2}}\sum_{l=0}^{N_{Fock}}{\frac{{\left(-1\right)}^{l}N_{Fock}!}{{\left(l!\right)}^{2}\left(N_{Fock}-l\right)!}}^{\zeta^{2l}}{\left(1-e^{-i\omega_{0}t}\right)}^{l}{\left(1-e^{i\hbar \omega_{0}t}\right)}^{l}e^{\zeta^{2}e^{-i\omega_{0}t}}\\ & \;=e^{-\zeta^{2}}\sum_{l=0}^{N_{Fock}}\frac{{\left(-1\right)}^{l}N_{Fock}!}{{\left(l!\right)}^{2}\left(N_{Fock}-l\right)!}\zeta^{2l}{\left(-1\right)}^{l}{\left(e^{i\omega_{0}t/2}-e^{-i\omega_{0}t/2}\right)}^{2l}e^{\zeta^{2}e^{-i\omega_{0}t}}\\ & \;=e^{-\zeta^{2}}\sum_{l=0}^{N_{Fock}}\frac{N_{Fock}!}{{\left(l!\right)}^{2}\left(N_{Fock}-l\right)!}\zeta^{2l}e^{il\omega_{0}t}{\left(1-e^{-i\omega_{0}t}\right)}^{2l}e^{\zeta^{2}e^{-i\omega_{0}t}}\\ & \;=e^{-g}\sum_{k=0}^{\infty }\sum_{l=0}^{N_{Fock}}\frac{{\left(-1\right)}^{k}N_{Fock}!}{{\left(l!\right)}^{2}\left(N_{Fock}-l\right)!}g^{l}e^{-i\left(k-l\right)\omega_{0}t}L_{k}^{2l-k}\left(g\right) \end{array}$$

Here, $g=\zeta^{2}$. In the last step, we have used the following equation [36]:

$${\left(1+\beta \right)}^{\alpha }e^{-\beta x}=\sum_{k=0}^{\infty }L_{k}^{\alpha -k}\left(x\right)\beta^{k}$$

Finally, the correlation function can be rewritten in following form:

(A3) $$\langle X\left(t\right)X^{†}\rangle_{Fock}=\sum_{k=0}^{\infty }\sum_{l=0}^{N_{Fock}}\Phi_{kl}e^{-i\left(k-l\right)\omega_{0}t}$$

where $\Phi_{kl}=e^{-g}\frac{{\left(-1\right)}^{k}N_{Fock}!g^{l}}{{\left(l!\right)}^{2}\left(N_{Fock}-l\right)!}L_{k}^{2l-k}\left(g\right)$.

## Appendix B. The Derivation of Equation (11)

Based on the Equation (A3), Equation (6) can be rewritten as:

(A4) $$G_{\sigma }^{r}(t)=\sum_{k=0}^{\infty }\sum_{l=0}^{N_{Fock}}\Phi_{kl}\left\{{\tilde{G}}_{\sigma }^{r}(t)e^{-(k-l)\omega_{0}t}+\theta (t){\tilde{G}}_{\sigma }^{<}(t)\left(e^{-(k-l)\omega_{0}t}-e^{(k-l)\omega_{0}t}\right)\right\}$$

After the Fourier transform, one obtains:

(A5) $$G_{\sigma }^{r}(\omega )=\sum_{k=0}^{\infty }\sum_{l=0}^{N_{Fock}}\Phi_{kl}\left\{{\tilde{G}}_{\sigma }^{r}(\omega -(k-l)\omega_{0})+\frac{1}{2}\left[{\tilde{G}}_{\sigma }^{<}(\omega -(k-l)\omega_{0})-{\tilde{G}}_{\sigma }^{<}(\omega +(k-l)\omega_{0})\right]\right\}$$

By the method of the equation of motion [26], under the limitation of U→∞, one can obtain:

$${\tilde{G}}_{\sigma }^{r}(\omega )=\frac{1-\langle n_{\bar{\sigma }}\rangle }{\hbar \omega -{\tilde{\varepsilon }}_{\sigma }-{\tilde{\Sigma }}_{0\sigma }^{r}(\hbar \omega )-{\tilde{\Sigma }}_{1\sigma }^{r}(\hbar \omega )}$$

Since Re(${\tilde{\Sigma }}_{0\sigma }^{r}$) due to the hopping between QD and leads has little influence on our qualitative results, we omit it here for convenience [25]. So, one gets ${\tilde{\Sigma }}_{0\sigma }^{r}=-i{\tilde{\Gamma }}_{\sigma }$. If we denote ${\tilde{\Sigma }}_{1\sigma }^{r}\left(\hbar \omega \right)=B_{\sigma }\left(\hbar \omega \right)+iA_{\sigma }\left(\hbar \omega \right)$, we can obtain Equation (11) from (A5) based on the following equation [26]:

(A6) $${\tilde{G}}_{\sigma }^{<}=i\left({\tilde{G}}_{\sigma }^{r}-{\tilde{G}}_{\sigma }^{a}\right){\bar{f}}_{\sigma }$$

## Appendix C. The Derivation of 〈X〉

Using the Baker-Campbell-Haussdorf relation, one can obtain:

(A7) $$X=\exp\left[-\zeta \left(a^{†}-a\right)\right]=exp\left(-g/2\right)\exp\left(-\zeta a^{†}\right)\exp\left(\zeta a\right)$$

And using the Equation (A1), we find:

(A8) $$\langle N_{Fock}\left|X\right|N_{Fock}\rangle =exp\left(-\frac{g}{2}\right)L_{N_{Fock}}\left(\zeta^{2}\right)=exp\left(-\frac{g}{2}\right)L_{N_{Fock}}\left(g\right)$$

which is for the Fock state.

As for our thermal state, one can find [35]:

(A9) $$\begin{array}{cc} {\langle X\rangle }_{thermal} & =\left(1-e^{-\beta \hbar \omega_{0}}\right)\sum_{N=0}^{\infty }e^{-\beta N\hbar \omega_{0}}\langle N|X|N\rangle \\ & =\left(1-e^{-\beta \hbar \omega_{0}}\right)exp\left(-\frac{g}{2}\right)\sum_{N=0}^{\infty }e^{-\beta N\hbar \omega_{0}}L_{N}\left(g\right) \end{array}$$

(A10) $${\langle X\rangle }_{thermal}=e^{-\left(N_{th}+1/2\right)g}$$

Here, we have used the generating function $\sum_{n=0}^{\infty }x^{n}L_{n}\left(g\right)=e^{-gx/\left(1-x\right)}/\left(1-x\right)$ in the last step, and $N_{th}=1/\left(e^{\beta \hbar \omega_{0}}-1\right)$.
